# Fabrication of Si Micropore and Graphene Nanohole Structures by Focused Ion Beam

**DOI:** 10.3390/s20061572

**Published:** 2020-03-12

**Authors:** Nik Noor Nabilah Md Ibrahim, Abdul Manaf Hashim

**Affiliations:** Malaysia Japan International Institute of Technology, Universiti Teknologi Malaysia, Jalan Sultan Yahya Petra, Kuala Lumpur 54100, Malaysia; nickcancer_nabiey@yahoo.com

**Keywords:** micropore, graphene, nanohole, focused ion beam, biosensor, DNA

## Abstract

A biosensor formed by a combination of silicon (Si) micropore and graphene nanohole technology is expected to act as a promising device structure to interrogate single molecule biopolymers, such as deoxyribonucleic acid (DNA). This paper reports a novel technique of using a focused ion beam (FIB) as a tool for direct fabrication of both conical-shaped micropore in Si_3_N_4_/Si and a nanohole in graphene to act as a fluidic channel and sensing membrane, respectively. The thinning of thick Si substrate down to 50 µm has been performed prior to a multi-step milling of the conical-shaped micropore with final pore size of 3 µm. A transfer of graphene onto the fabricated conical-shaped micropore with little or no defect was successfully achieved using a newly developed all-dry transfer method. A circular shape graphene nanohole with diameter of about 30 nm was successfully obtained at beam exposure time of 0.1 s. This study opens a breakthrough in fabricating an integrated graphene nanohole and conical-shaped Si micropore structure for biosensor applications.

## 1. Introduction

Biosensors have attracted a great deal of attention for biological detection in the past three decades [1]; for example, DNA biosensors are of major interest owing to their great promise for obtaining sequence-specific information in a faster, simpler and cheaper manner [2,3,4,5,6]. DNA sequencing on a single molecule basis is extremely useful in medical fields for diagnosis and personalized medicine. A solid-state biosensor based on a micropore structure has been considered as a promising structure to interrogate single molecule detection [7,8]. The first biosensor of this kind was successfully demonstrated in 1996, using α-haemolysin as a sensing membrane to detect DNA molecules [6,9,10,11]. However, those biomaterial sensing membranes are subjected to denaturation and degradation after some time of being in use due to several influencing factors such as pH, temperature, corrosive chemicals [12,13,14,15,16,17], and so forth. Moreover, the size of the fabricated biological channel is hard to control, even though various modifications are applied, mainly through so-called protein engineering, etc. [17]. 

Graphene, a two-dimensional nanomaterial, facilitated with a nanohole and integrated onto a solid-state micropore structure is considered as a promising alternative to realize a stable biosensor due to its one-atom thick layer and its extremely high surface-to-volume ratio, which enable it to be used as a highly sensitive membrane for the detection of biomaterials, such as DNA and protein [18,19,20,21,22,23]. Merchant et al. reported that using a graphene nanopore membrane mounted on a SiN micropore could increase signal amplitude during the translocation of DNA through a graphene nanopore [22]. Here, the fabrication processes of the graphene biosensor involve multiple difficult steps, such as dry etching, electron beam lithography and a deposition process, causing pinholes in the graphene membrane, which affect the ionic current signal [22]. Goyal et al. showed the detection of protein through graphene nanopore on a SiN fabricated micropore by transmission electron microscopy (TEM) [23]. However, graphene tends to show a shrinking behavior after a milling process by TEM. In this work, we propose to utilize a focused ion beam (FIB) system to directly fabricate both conical-shaped micropore in a Si substrate and a nanohole in graphene after being transferred onto a Si micropore substrate. We expect that the major advantage of using a FIB is the excellent controllability of size and shape of both conical-shaped Si micropores and the graphene nanoholes. Here, it is important to note that a conical-shaped Si micropore acts as the fluidic channel for the targeted sensing elements, while graphene acts as the sensing membrane. It was reported that a fluidic channel with a conical-shaped structure could realize a dramatic increase in the transportation of sensing elements, as compared to a cylindrical-shaped pore structure due to a low transporting resistance [24,25]. In addition, it was reported that a major challenge in realizing high-resolution DNA sequencing with micropore structure is the finite length of the sensing channel constituting the pore structure [26]. The typical solid-state fluidic channel with micropore structures is thick, resulting in a large number of bases (~100) present in the porous membrane [26]. Wanunu et al. [24] reported that reducing the thickness of the micropore structure would lead to an increase in signal amplitudes from biomolecules [24,27]. In this paper, first we report the fabrication of a conical-shaped micropore structure by utilizing a combination of grinding and polishing to thin the Si substrate, followed by a multi-milling by the FIB system. Then, we demonstrate the transfer step of graphene onto the Si micropore by using an all-dry transfer method in order to minimize the contamination on the graphene surface. Finally, we report the fabrication of the nanohole in graphene by the FIB. 

## 2. Materials and Methods

**Figure 1:**
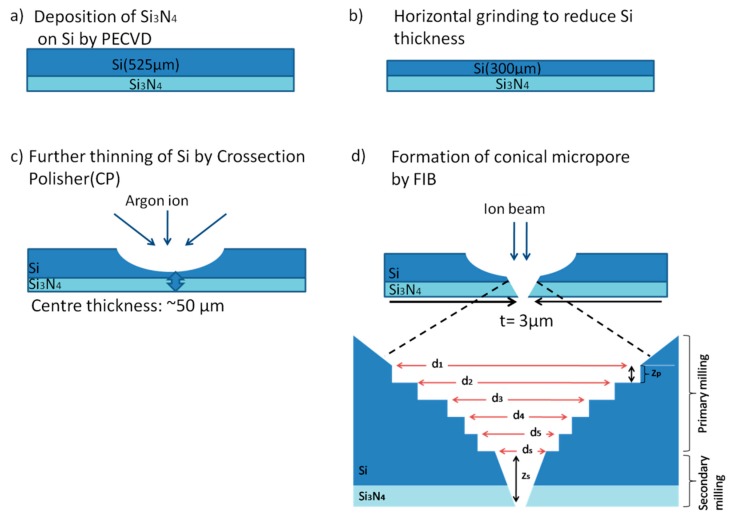
Fabrication process of the conical-shaped Si micropore structure, (**a**) deposition of Si_3_N_4_ on Si, (**b**) horizontal grinding of Si, (**c**) thinning of Si, and (**d**) milling of substrate.

At first, the Si substrate (thickness: 525 µm) was cleaned with conventional organic treatment (acetone, ethanol and deionized (DI) water) to remove native oxides and contaminants on the substrate surface. Figure 1 summarizes the fabrication steps used in this work to obtain conical-shaped micropore structure. 

Silicon nitride (Si_3_N_4_) was deposited onto a Si substrate with a thickness of 100 nm using plasma-enhanced chemical vapor deposition (PECVD), as shown in Figure 1a. Then, a horizontal grinding was applied to reduce the thickness of the Si substrate down to ~300 µm as shown in Figure 1b, followed by a vertical polishing process using an argon ion polisher to further thin down the area of the micropore formation to be around ~50 µm, as shown in Figure 1c, so that the subsequent milling by the FIB can be done in a short time. The combination of grinding and polishing processes in such a way was needed in order to achieve such targeted thickness and to avoid the possibility of cracking, especially during the transfer process of the graphene. After that, a multi-step milling using the FIB system (FEI Quanta 3D 200i) was introduced in order to obtain a conical-shaped micropore, as shown in Figure 1d. Specifically, this multi-step milling consists of primary and secondary milling. Here, the ion beam currents, *I*, were set at 2.1 and 9.3 nA for primary and secondary milling, while the ion beam voltage, *V*, was kept constant at 30 kV. It can be seen here that the primary milling was performed in a multistep manner where the milling diameter was changed at every milling step, as the upper diameter is always set larger than the bottom diameter so that the conical-shape of the micropore can be realized. At the primary milling, the diameter was changed in the condition that can be expressed as d_1_ > d_2_ > d_3_ >….> d_n_. The milling diameter was changed from 40 down to 15 µm, with the 5 µm in difference. Each milling step was carried out at the same depth value, z_p_, which is about 3 µm in depth. At the secondary milling stage, the milling was carried out in a single step. The value for d_s_ and z_s_ were always fixed to 3 and 1000 µm, respectively. The substrate with the micropore structure was characterized using scanning electron microscopy (SEM). 

**Figure 2:**
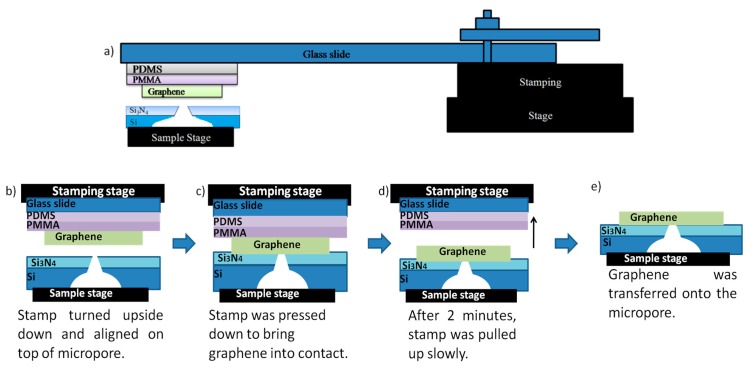
Schematic of the all-dry transfer method of graphene onto the Si micropore (**a**) the micromanipulator was used to adjust the position of the graphene on the micropore (**b**) stamping and sample stage structures were prepared (**c**) graphene was brought into contact with the micropore (**d**) and (**e**) stamping stage was retracted with graphene left on the micropore.

A mechanically exfoliated graphene was used in this study. The obtained graphene is firstly transferred onto the PMMA/PDMS layer, coated on a glass slide prior to the subsequent all-dry transfer process to locate the graphene on the micropore as shown in Figure 2. Figure 2 summarizes the steps of the all-dry transfer method of the graphene layer onto the Si micropore. 

This all-dry transfer process was carried out using an optical microscope facilitated with a micromanipulator (Figure 2a), which consisted of a stamping and a sample stage, placed facing each other. The stamping stage was constructed with graphene/PMMA/PDMS/glass slide and was aligned facing towards the micropore as shown in Figure 2b. As shown in Figure 2c, graphene was brought into contact with a micropore by slowly pressing down the stamping stage. Graphene was kept in contact with the micropore for 2 min before the stamping stage was retracted, leaving the graphene on the micropore as shown in Figure 2d. The graphene layer was confirmed to attach to the micropore without any PMMA residue as shown in Figure 2e. It can be understood that such a direct transfer method does not involve any chemical solvents and it is expected that a quality of graphene with no defect or contaminant can be realized for better device performance.

Finally, a nanohole was formed in the transferred graphene on the Si micropore by the FIB at a constant beam voltage and current of 30 kV and 1.1 pA, respectively. The dependence of exposure time was in the range of 1 to 2.4 s with a 0.2 s interval time. The nanohole diameter and shape was characterized using scanning electron microscopy (SEM—Helios Nanolab G3) and Raman spectroscopy (Witec Alpha 300 M+).

## 3. Results

**Figure 3:**
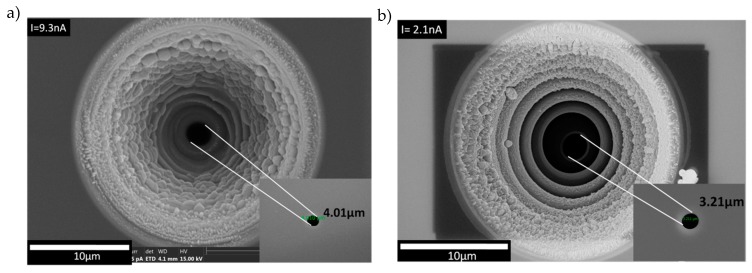

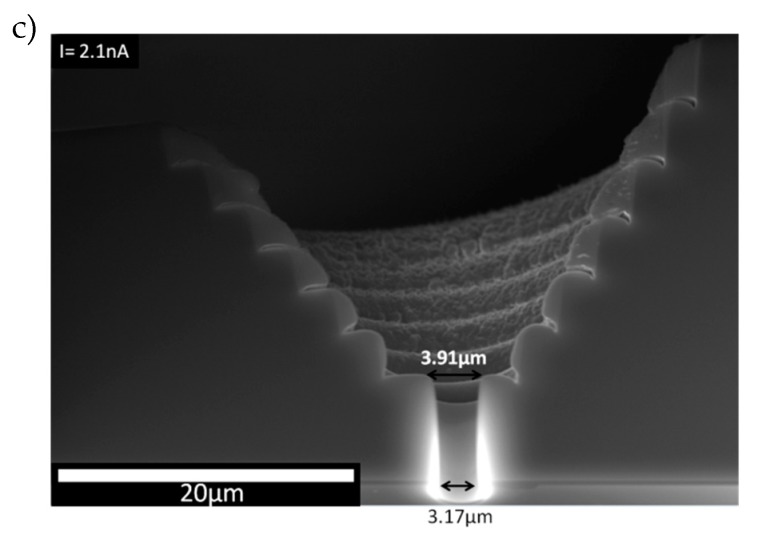
Top and bottom view (inset) SEM images of the micropore fabricated at a milling current of (**a**) 9.3 nA and (**b**) 2.1 nA. (**c**) cross-sectional view of the conical-shaped micropore structure milled at 2.1 nA.

For the results, first we present the dependency of the Si micropore’s shape and size with the change of the FIB milling current. Then, the dependency of the shape and size of the graphene nanohole as a function of the FIB milling time is described. The properties of the transferred graphene membrane on the Si micropore using the all-dry transfer technique is also presented. Figure 3a,b show both the top and bottom views (inset) of SEM images of the Si micropore milled at milling current, *I*, of 9.3 and 2.1 nA, respectively. As shown in the Figure 3a,b, significant differences in hole shape and size can be observed. A milling at higher current of 9.3 nA seems to produce an uneven diameter and a larger size of pore as compared to the one milled at a low milling current of 2.1 nA. This happens due to the larger beam current corresponding to the large beam spot size [28]. Figure 3c shows the cross-sectional image of the conical- shape micropore milled also at 2.1 nA. Here, it can be confirmed that a well-defined conical-shaped micropore structure was obtained. From Figure 3b,c, it can be understood that those two different samples milled at the same milling current of 2.1 nA produce a relatively consistent micropore diameter at the bottom side (3.21 µm and 3.17 µm). In addition, it can be seen that the effect of a so-called redeposition of Si takes place during the milling, where the sputtered Si was deposited back onto the surface [29]. Again, it can be said that a lower milling current tends to suppress the redeposition effect, where it produces a well-defined circular-shaped hole. A through hole with one step milling at the secondary milling was successfully obtained with a diameter value that is close to the setting value. From this observation, we can conclude that the combination of primary and secondary milling by the FIB at a lower milling current will lead to a better geometrical accuracy and a smaller redeposition effect.

**Figure 4:**
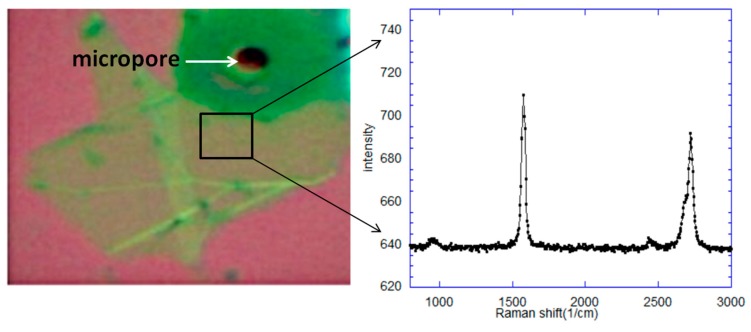
Optical image and Raman spectra of graphene transferred onto the Si micropore.

Figure 4 shows the graphene transferred onto the conical-shaped micropore using the all-dry transfer process. As shown in Figure 4, the Raman spectra positions of G and 2D peak at 1580 cm^−1^ and 2690 cm^−1^, respectively [30]. The intensity ratio of I_2D_/I_G_ of 1.1 indicates that the transferred graphene layer is a bilayer graphene [31]. No defect or D peak is detected at ~1348 cm^−1^, which indicates that clean and defect free graphene was successfully transferred by the all-dry transfer method.

**Figure 5:**
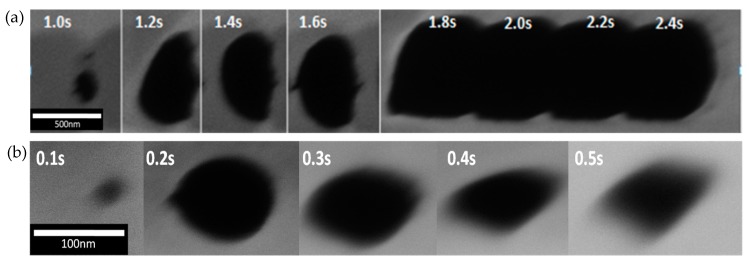
(**a**) the changes of hole size and shape with milling time from 1.0 to 2.4 s, (**b**) the changes of hole size and shape with milling time from 0.1 to 0.5 s.

The formation of the nanohole was performed in the transferred graphene on the Si micropore by a direct milling using the FIB. Figure 5a shows the changes of the hole size and shape as a function of milling time. It can be seen that the size increases with the milling time. We can see that the coalescence of the holes starts to take place from 1.8 s. This deformation occurs probably due to the coupled effect of atomic displacement and electronic energy displacement from ion-solid material interaction [32]. Figure 5b shows the size of the graphene holes when the milling time is reduced to be below 0.5 s in order to obtain a smaller size and a well-defined circular-shaped nanohole. A circular-shaped nanohole with a diameter of 30 nm has been successfully obtained at a milling time of 0.1 s.

**Figure 6:**
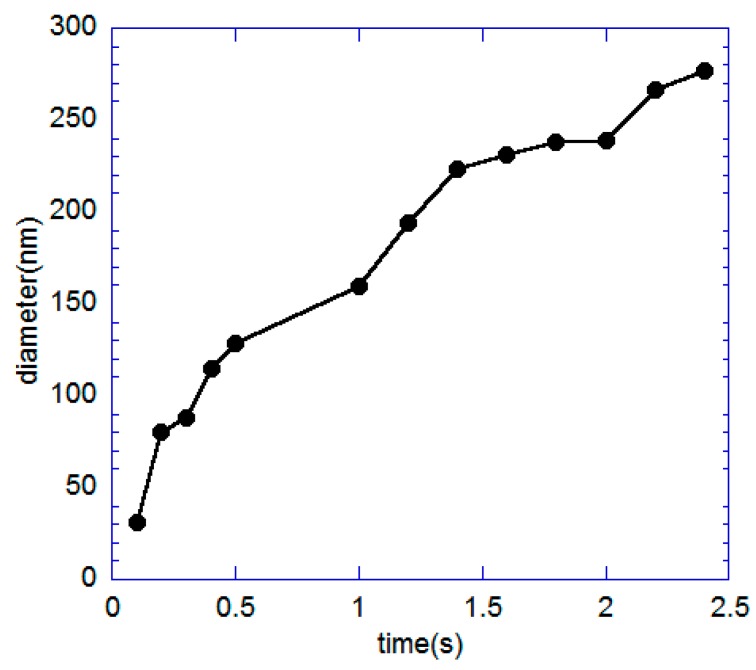
The diameter of the nanohole with the change of milling time.

Figure 6 plots the changes of the graphene nanohole diameter as a function of milling time where it shows an almost linear change. Here, it can be said that the FIB is one of the most reliable direct milling techniques to create micropore and nanohole by applying very minimum control parameters.

**Figure 7:**
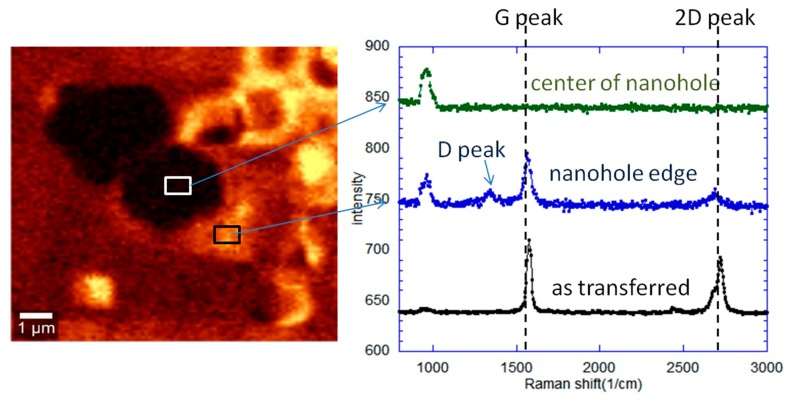
Changes in Raman spectra of graphene at the center of the nanohole and graphene edge.

The Raman spectra was measured to confirm the nanohole formation in the graphene layer. Figure 7 shows the Raman spectra measured at the center of the micropore and at the area near to the micropore. No G and 2D peak are observed at the center of the micropore, which seems to confirm that the hole was created. Meanwhile, graphene with a defect was observed at the area near to the micropore, confirming the existence of graphene. It can be speculated that the appearance of this small defect peak was probably due to the side effect of milling.

## 4. Conclusions

In summary, a simple and reliable direct fabrication of a conical-shaped Si micropore and a graphene nanohole using a FIB was successfully demonstrated for the first time. A clean and defect free graphene was also successfully transferred accurately onto a micropore by a developed all-dry transfer method. This study opens a breakthrough towards the utilization of integrated graphene nanohole and conical-shaped Si micropore for biosensor applications.
